# Spinal pain prevalence and associated determinants: A population‐based study using the National Survey for Wales

**DOI:** 10.14814/phy2.70101

**Published:** 2024-10-29

**Authors:** David C. Byfield, Benjamin S. Stacey, Hywel T. Evans, Ian W. Farr, Leon Yandle, Lora Roberts, Teresa Filipponi, Damian M. Bailey

**Affiliations:** ^1^ Neurovascular Research Laboratory, Faculty of Life Sciences and Education University of South Wales Pontypridd UK; ^2^ SAIL Databank, Population Data Science Swansea University Medical School Swansea Wales UK

**Keywords:** comorbidities, deprivation, physical inactivity, prevalence, socioeconomics, spinal pain

## Abstract

Spinal pain (SP) remains the leading cause of disability worldwide. The present study aimed to establish a current prevalence of SP and associated determinants in Wales by retrospectively analyzing data from the National Survey for Wales Dataset (NSWD). The NSWD is a large‐scale cross‐sectional, representative sample of adults across Wales, UK. A univariable and multivariable regression analysis was carried out on self‐reported answers to health and well‐being questions contained within the NSWD (2016–2020) to determine the strength of association of various determinants and comorbidities related to spinal pain. A total population of 38,954 of adults were included in the analysis. The study population included interview responses of 21,735 females and 17,219 males. The prevalence of SP in Wales was 4.95% (95% CI: 4.74%–5.15%) with a total of 847 males (4.92%, CI: 4.60%–5.24%) and 1082 females (4.98%, CI: 4.69%–5.27%) reporting spinal pain. The age group with the highest prevalence of SP was in the 70+ years age group for both males (5.44%, CI: 4.82%–6.07%) and females (5.95%, CI: 5.37%–6.54%). The strength of association between age and SP reaches its peak at 50–59 years with an adjusted Odds Ratio (aOR) of 3.74 (*p* = <0.001), that decreases slightly at 60–69 years and 70+ years. For various comorbidities included in the NSWD, significant associations with SP were confirmed for: mental illness (aOR = 1.42, *p* = <0.001), migraine (aOR = 2.73, *p* = <0.001), nervous system issues (aOR = 1.61, *p* = <0.001), arthritis (aOR = 1.30, *p =* <0.001) and issues with bones/joints/muscles (aOR = 1.93, *p =* <0.001). For lifestyle factors, associations were confirmed for current smokers (aOR = 1.41, *p* = <0.001) and ex‐smokers (aOR = 1.23, *p* = 0.003). This study demonstrates a low prevalence of SP in Wales when compared to global estimates and strong associations to a variety of determinants. This still represents a significant societal burden and these findings may help inform public health initiatives to encourage prevention and evidence‐based interventional strategies and ultimately, improve the quality of life for those suffering with SP in Wales.

## 1 INTRODUCTION

Collectively referring to neck pain (NP) and/or back pain (BP), spinal pain (SP) remains the leading cause of global disability worldwide (Buchbinder et al., 2020; Ferreira & de Luca, 2017; GBD 2021 Low Back Pain Collaborators, 2023; Hartvigsen et al., 2018; Wu et al., 2020) and is recognized as a complex condition underpinned by a number of psychological, social and biological factors (Hartvigsen et al., 2018; Hoy et al., 2012). With a lifetime prevalence rate estimated at 70% (Foster et al., 2018; Ramanathan et al., 2018; Rundell et al., 2017; Williams et al., 2018), SP has also been closely linked with several comorbidities, including type 2 diabetes, obesity, cardiovascular disease, and neurodegenerative diseases (de Luca et al., 2023; Fernandez et al., 2016, 2017; Whitlock et al., 2017). This is likely attributed to the disability associated with SP predisposing patients to a more sedentary lifestyle (da Cruz Fernandes et al., 2018; Jonsdottir et al., 2019; Scarabottolo et al., 2017)—a primary determinant contributing to a rise in chronic ill health in later life (Bailey et al., 2013; Gallaway et al., 2017; Marley et al., 2020). Additionally, poor lifestyle choices such as smoking, excessive alcohol consumption and low physical activity, also appears to contribute to persistent SP and a reduction in overall health (Jonsdottir et al., 2019; Williams et al., 2018).

Recent evidence has indicated that females exhibit a higher age‐standardized prevalence of SP compared to males globally, which may be geographically dependent (Wu et al., 2020). There is also evidence that SP and related comorbidities affect older people with reduced capacity and overall quality of life (Hartvigsen et al., 2018; Pinto et al., 2023; Schneider et al., 2006). The severity and prevalence of SP has also been observed to be much higher in those with a low educational attainment and in areas of greater deprivation (Jonsdottir et al., 2019; Volkers et al., 2007). Those living in more deprived areas experience accelerated transitions to multiple chronic disease(s) and early death across all ages, which is particularly evident in the Welsh population (Lyons et al., 2023). The people of Wales exhibit a disproportionally elevated burden of cardio/neurovascular disease, attributed primarily to higher levels of physical inactivity, obesity, comorbidities, alcohol consumption and smoking, negatively impacting quality of life compared with other UK nations (Scarborough et al., 2011) and European states (Steel et al., 2018). It has also been reported that cardiorespiratory fitness is excessively low in the Welsh population both in (vascular) disease‐free controls and diseased patients outlining the poor health status in the country (Bailey et al., 2024; Lanéelle et al., 2024).

A previous study utilisising the Welsh Health Survey (WHS) reported a prevalence of SP to be as high as 39%, taking into account those reporting both acute (32%) and chronic (13%) pain (Jonsdottir et al., 2019). However, the 2012 WHS employed a checklist and classified acute back pain as untreated back pain in the past 12 months and chronic pain as back pain currently being treated, which may have overestimated SP prevalence in Wales. Therefore, the present study aimed to quantify and provide a comprehensive update of the prevalence of SP and the association of various determinants (please refer to the Variables section in the Materials and Methods for all determinants), using an open‐question format of the health‐related questions from the National Survey for Wales Dataset (NSWD).

## 2 MATERIALS AND METHODS

### 2.1 Ethical approval

Ethical approval was granted by the Faculty of Life Sciences and Education Research Ethics Committee at the University of South Wales (#210607LR) with data governance approval granted by the SAIL Databank Information Governance Review Panel (SAIL Project 1315). Paticipants have consented to take part in the National Survey for Wales which includes consent for linkage.

### 2.2 Design and data sources

The study was a cross‐sectional population‐based observational study conducted in accordance with the published guidelines outlined in the most recemt version of the STROBE statement guidelines for reporting observational studies (von Elm et al., 2014). Data from the NSWD were obtained via the Secure Anonymised Information Linkage (SAIL) Databank (Ford et al., 2009; Jones et al., 2019; Lyons et al., 2009), a secure repository containing anonymised health and administrative data about the population of Wales. Individual‐level linkage was made to the Welsh Demographic Service Dataset (WDSD), which contained demographic information for 83% of people registered with a general practice (GP) in Wales. Using the WDSD provided the lower‐layer super output area (LSOA) 2011 code of the participant's address at the time of the survey, which allowed for the determination of the Welsh Index of Multiple Deprivation (WIMD) 2019 quintile for the LSOA (Welsh Index of Multiple Deprivation|GOV.WALES, n.d.). LSOAs are geographic units designed for the reporting of small area statistics and were aggregated to report results by Welsh local authority. Age was also extracted from WDSD, which was useful for reflecting relevant milestones across the lifespan (young adult, working age, retirement age and older age).

### 2.3 National Survey for Wales dataset (NSWD)—Data source

#### 2.3.1 Study setting

The NSWD is a large‐scale cross‐sectional, representative sample of adults across all jurisdictions of Wales where participants are interviewed by the Office for National Statistics and provide self‐report answers to health and well‐being questions (Welsh Government, 2023).

#### 2.3.2 Participants

**FIGURE 1:**
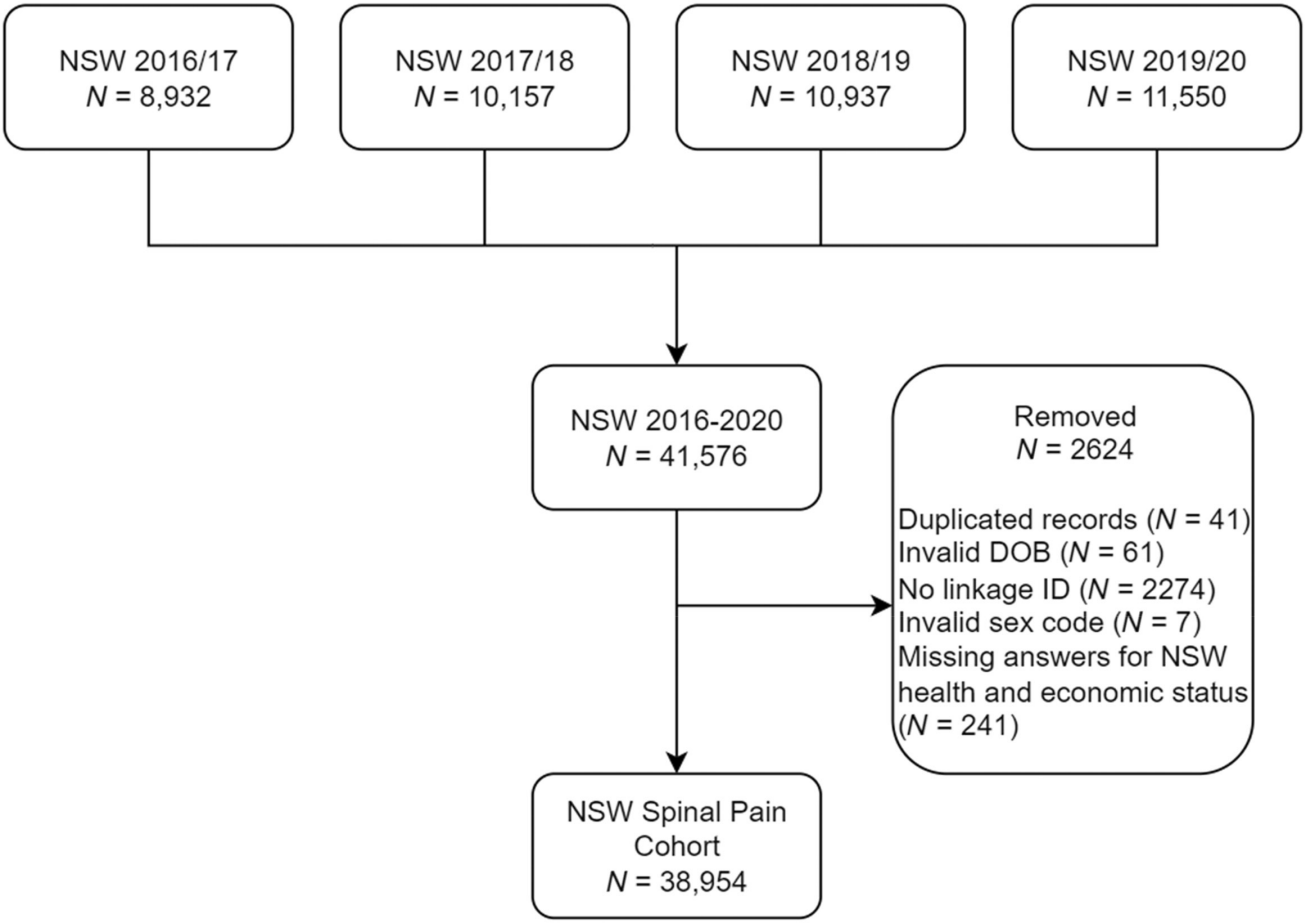
Flowchart of the National Survey for Wales Dataset (NSWD) population selection for the study analysis (2016–2020). The SAIL Databank held information for around 83% of the Welsh population, which accounts for the excluded cases without a linkage ID.

Participants from 2016/17 to 2019/20 and aged over 16 years old were included in the analysis. Individuals with an invalid date of birth, missing ID code and sex, and unanswered NSWD health or economic status questions were removed, as illustrated in Figure 1. For participants who appeared in multiple years of the NSWD, only their first record was retained and analyzed. We chose this data sampling period to ensure that the data collection methods were consistent in order to collate the data longitudinally. The data collection methodology changed commencing during the covid‐19 pandemic (2021–22) using telephone interviews.

#### 2.3.3 Variables

As part of the NSWD survey, participants were specifically asked an open general health question (not a checklist), “Do you have any physical or mental health conditions or illnesses lasting or expected to last for 12 months or more?”. If Yes, participants were asked, “What other health condition or illness do you have?”, to allow for multiple conditions to be recorded. Therefore, survey respondents were asked to spontaneously identify the conditions affecting them, rather than being prompted by a check list focussing on longstanding illness and conditions. The primary outcome for this study was SP prevalence, which was defined as a participant self‐reporting any of the following: back, slipped disc, spine, and neck pain, or being limited by these problems.

Data for the following determinants were explored for associations with spinal pain: *Participant demographics*: age groups (16–29, 30–39, 40–49, 50–59, 60–69, 70+ years); biological sex (male, female); *Socioeconomics*: self‐reported highest educational attainment; Body Mass Index (BMI) category; WIMD quintile (of residence at time of NSWD survey), which considers the following: income, employment, health, education, access to services, housing, community safety, and physical environment (“WIMD Index Guidance”, 2019) (Welsh Index of Multiple Deprivation 2014, n.d.); smoking status (currently smoking, ex‐smoker, or never smoked); alcohol category (usual weekly consumption: non‐drinker, lower risk (≤ 14 units per week), higher risk (> 14 units per week)); economic status; physical activity (≥ 150 min of moderate to vigorous physical activity per week), as per World Health Organization (WHO) (DiPietro et al., 2020), physical inactivity (< 30 min of moderate to vigorous physical activity per week). We also sought to establish the relationship with *multicomorbidities* to better understand the wider impact of/on spinal pain, including: cancer; diabetes (including hyperglycaemia); migraine headaches; mental illness, anxiety, depression; stroke, cerebral hemorrhage, cerebral thrombosis; heart attack, angina; other problems of the nervous system; hypertension; emphysema; asthma, arthritis, rheumatism, fibrositis; other problems of bones, joints and muscles; heart and circulatory illness; respiratory system illness; musculoskeletal illness.

#### 2.3.4 Statistical analysis

Descriptive statistics were presented using tabulated counts of all participants, which were stratified by SP, a binary outcome variable as defined in the Variables section, for each determinant. This was used to calculate prevalence, defined as the number of participants reporting SP divided by the total number of respondents stratified by sex, in combination with 95% confidence intervals (CI) using the Wald Method (Agresti, 2007). Spatial mapping was perfomed to demonstrate SP prevalence by individual Welsh local authority. The strength of association between the outcome and the determinants were quantified by cross‐sectional analysis to obtain odds ratios (ORs) for: univariable (crude) logistic regression for each determinant (cORs), and multivariable regression (fully adjusted) for all determinants (aORs). Reference categories for each variable can be seen in Table A1. All statistical analyses were conducted using R v4.1.3 (R Core Team, R Foundation for Statistical Computing: Vienna, Austria).

## 3 RESULTS

### 3.1 Study population

A total population cohort of 38,954 participants from the NSWD were analyzed in the study, following the exclusion of 2622 participants (Figure 1), representing a sample of 1.3% of the Welsh population (Office for National Statistics, 2022). The study population included responses by 21,735 (55.8%) females and 17,219 (44.2%) males with demographics for the study population summarized in Table 1. After linkage to WDSD, the most deprived WIMD quintile was the least represented, comprising 16.8% of the cohort (compared to 20% in the total Welsh population, by definition).

**TABLE 1:** Prevalence of spinal pain and predictors.

Predictors		*n* (% of cohort)	No SP	SP	SP male (% of male cohort)	SP female (% of female cohort)
Total cohort		38,954				
Spinal Pain	No spinal pain	37,025 (95.0)				
	Spinal pain	1929 (5.0)				
Sex	Male	17,219 (44.2)	16,372 (44.2)	847 (43.9)	847 (100)	
	Female	21,735 (55.8)	20,653 (55.8)	1082 (56.1)		1082 (100)
Age (years)	16–29	3683 (9.5)	3635 (98.7)	58 (1.5)	21 (1.3)	37 (1.8)
	30–39	4964 (12.7)	4818 (97.0)	146 (2.9)	61 (2.0)	85 (2.9)
	40–49	5067 (13.0)	4820 (95.1)	247 (4.9)	110 (4.9)	137 (4.8)
	50–59	6502 (16.7)	6116 (94.0)	386 (5.9)	173 (5.9)	213 (6.0)
	60–69	7383 (19.0)	6941 (94.0)	442 (6.0)	205 (2.7)	237 (5.9)
	70+	11,355 (29.1)	10,705 (94.0)	650 (5.7)	277 (5.4)	373 (6.0)
WIMD	5—Least deprived	8259 (21.2)	7922 (96.0)	337 (4.0)	149 (4.0)	188 (4.2)
	4	8484 (21.8)	8104 (96.0)	380 (4.5)	176 (4.6)	204 (4.4)
	3	8367 (21.5)	7954 (95.0)	413 (4.9)	180 (4.9)	233 (5.0)
	2	7284 (18.7)	6876 (94.3)	408 (5.6)	175 (5.5)	233 (5.7)
	1—Most deprived	6560 (16.8)	6169 (94.0)	392 (6.0)	167 (3.6)	224 (5.9)
Education	NQF levels 4–8	14,558 (37.4)	14,001 (96.2)	557 (3.8)	257 (3.9)	300 (3.8)
	NQF level 3	4779 (12.3)	4541 (95.0)	238 (5.0)	139 (5.3)	99 (4.6)
	NQF level 2	6631 (17.0)	6345 (95.7)	286 (4.3)	104 (4.2)	182 (4.4)
	Below NQF level 2	1401 (3.6)	1330 (95.0)	71 (5.1)	29 (4.9)	42 (5.2)
	No qualifications	9659 (24.8)	9006 (93.2)	653 (6.7)	267 (6.5)	386 (6.9)
Economic status	Employed	18,253 (46.9)	17,656 (96.7)	597 (3.3)	296 (3.2)	301 (3.1)
	Unemployed	895 (2.3)	845 (94.4)	50 (5.6)	24 (5.1)	26 (6.1)
	Economically inactive	19,806 (50.8)	18,524 (93.5)	1282 (6.5)	527 (6.4)	755 (6.5)
BMI	Healthy weight (18.5–24.9 kg/m^2^)	8195 (21.0)	7885 (96.2)	310 (3.8)	115 (2.3)	195 (3.9)
	Underweight (< 18.5 kg/m^2^)	375 (1.0)	360 (96.0)	15 (4,0)	5 (5.3)	10 (3.6)
	Overweight (25–29.9 kg/m^2^)	7985 (20.5)	7545 (94.4)	440 (5.5)	230 (5.4)	215 (5.7)
	Obese (>30 kg/m^2^)	5230 (13.4)	4860 (93.0)	370 (7.1)	165 (6.8)	200 (7.1)
Physical activity	Inactive	11,744 (30.1)	10,942 (93.2)	802 (6.8)	326 (7.8)	476 (6.8)
	Active	12,018 (30.9)	11,596 (96.5)	422 (3.5)	202 (3.6)	220 (3.4)
Smoker	Never smoked	11,647 (29.9)	11,171 (96.0)	476 (4.1)	162 (3.5)	314 (4.5)
	Ex‐smoker	7766 (19.9)	7312 (94.2)	454 (5.8)	227 (6.0)	227 (5.7)
	Current smoker	4345 (11.2)	4051 (93.2)	294 (6.8)	139 (7.1)	155 (6.5)
Alcohol	None	5148 (13.2)	4834 (94.0)	314 (6.1)	126 (6.7)	188 (5.7)
	Low risk	13,932 (35.8)	13,233 (95.0)	699 (5.0)	269 (4.8)	430 (5.1)
	High risk	4446 (11.4)	4239 (95.0)	207 (4.7)	133 (4.7)	74 (6.8)
Cancer	No	37,885 (97.3)	36,023 (95.1)	1862 (4.9)	819 (4.9)	1043 (4.9)
	Yes	1069 (2.7)	1002 (94.0)	67 (6.3)	28 (5.4)	39 (7.0)
Diabetes	No	36,435 (93.5)	34,666 (95.1)	1769 (4.9)	749 (4.7)	1020 (5.0)
	Yes	2519 (6.5)	2359 (94.0)	160 (6.4)	98 (7.2)	62 (5.3)
Mental illness	No	35,220 (90.4)	33,601 (95.4)	1619 (4.6)	727 (4.6)	892 (4.6)
	Yes	3734 (9.6)	3424 (92.0)	310 (8.3)	120 (8.6)	190 (8.1)
Migraine	No	38,726 (99.4)	36,828 (95.0)	1898 (4.9)	840 (4.9)	1058 (4.9)
	Yes	228 (0.6)	197 (86.4)	31 (13.6)	7 (11.3)	24 (14.5)
Nervous system	No	37,704 (96.8)	35,910 (95.0)	1794 (4.8)	788 (4.7)	1006 (5.0)
	Yes	1250 (3.2)	1115 (89.2)	135 (11.0)	59 (11.4)	76 (10.4)
Stroke	No	38,505 (98.8)	36,607 (95.1)	1898 (4.6)	832 (4.9)	1066 (5.0)
	Yes	449 (1.2)	418 (93.0)	31 (6.9)	15 (6.1)	16 (7.9)
Heart attack	No	38,305 (98.3)	36,435 (95.0)	1870 (4.9)	815 (4.8)	1055 (4.9)
	Yes	649 (1.7)	590 (91.0)	59 (9.1)	32 (8.7)	27 (9.6)
Hypertension	No	35,757 (91.8)	34,012 (95.1)	1745 (4.9)	756 (2.1)	989 (4.9)
	Yes	3197 (8.2)	3013 (94.2)	184 (5.8)	91 (6.1)	93 (5.5)
Emphysema	No	38,019 (97.6)	36,156 (95.1)	1863 (4.9)	806 (4.8)	1057 (5.0)
	Yes	935 (2.4)	869 (93.0)	66 (7.1)	41 (9.4)	25 (5.0)
Asthma	No	36,812 (94.5)	35,009 (95.1)	1803 (4.9)	807 (4.9)	996 (4.9)
	Yes	2142 (5.5)	2016 (94.1)	126 (5.9)	40 (5.0)	86 (6.4)
Arthritis	No	34,125 (87.6)	32,599 (95.5)	1526 (4.5)	697 (4.4)	829 (4.5)
	Yes	4829 (12.4)	4426 (92.0)	403 (8.3)	150 (10.2)	253 (7.5)
Bones, joints, muscles	No	36,184 (92.9)	34,555 (95.4)	1629 (4.5)	723 (4.5)	906 (4.5)
	Yes	2770 (7.1)	2470 (89.0)	300 (11.0)	124 (11.2)	176 (10.6)
Heart illness	No	32,418 (83.2)	30,905 (95.3)	1513 (4.7)	647 (4.6)	866 (4.7)
	Yes	6536 (16.8)	6120 (94.0)	416 (6.4)	200 (6.2)	216 (6.5)
Respiratory illness	No	35,394 (90.9)	33,698 (95.2)	1696 (4.8)	742 (4.7)	954 (4.8)
	Yes	3560 (9.1)	3327 (93.4)	233 (6.5)	105 (6.8)	128 (6.3)
Musculoskeletal illness	No	30,610 (78.6)	30,610 (0.0)	0 (0.0)	0 (0.0)	0 (0.0)
	Yes	8344 (21.4)	6415 (76.9)	1929 (23.1)	847 (27.9)	1082 (20.4)
Musculoskeletal limiting	No	31,486 (80.8)	31,330 (99.5)	156 (0.5)	68 (0.5)	88 (0.5)
	Yes	7468 (19.2)	5695 (76.3)	1773 (23.7)	779 (28.6)	994 (21.0)

Abbreviations: BMI, Body Mass Index; NQF, National Qualifications Framework; SP, Spinal Pain; WIMD, Welsh Index of Multiple Deprivation.

### 3.2 Spinal pain prevalence

**FIGURE 2:**
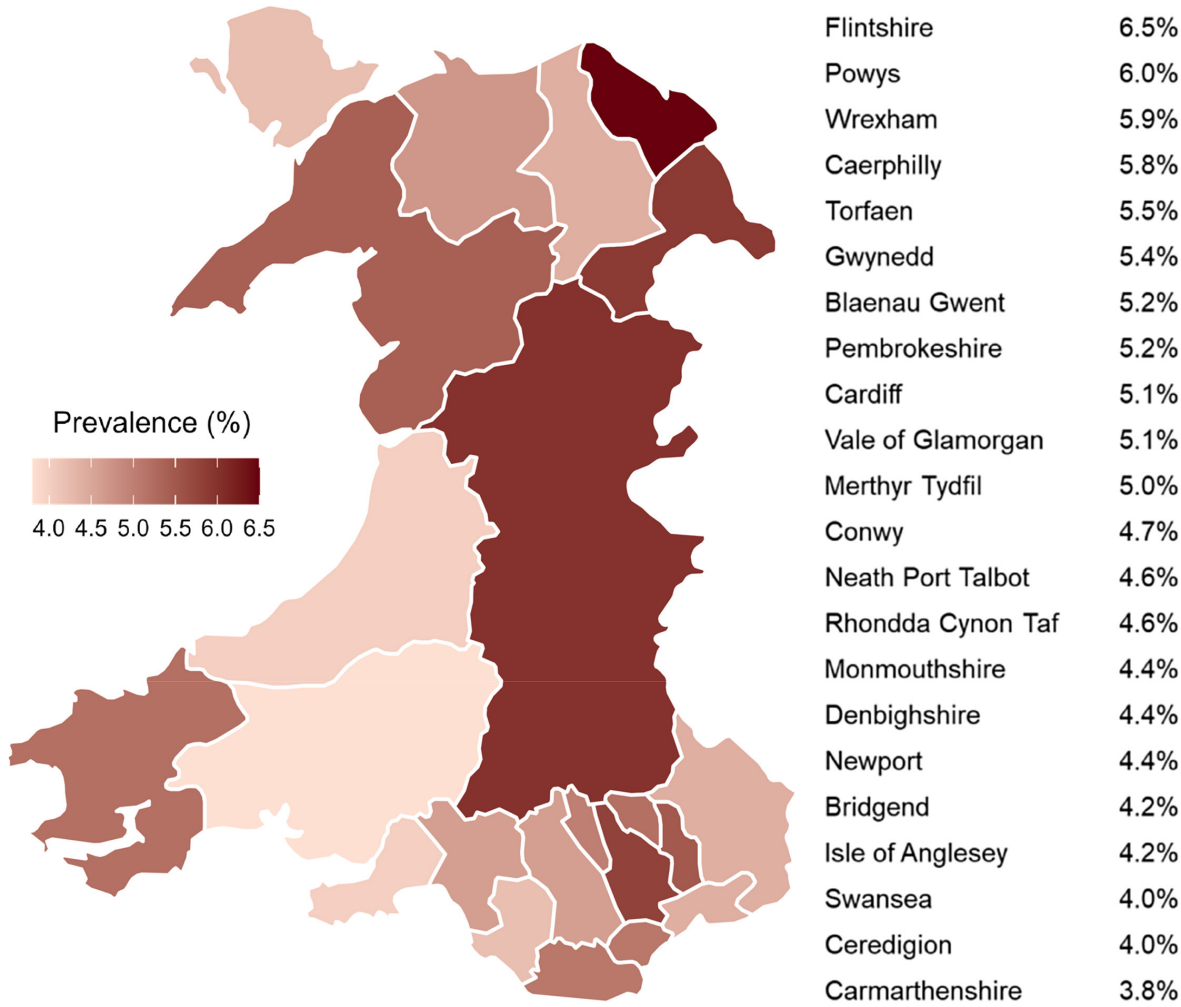
Heat Map of illustrating spinal pain prevalence by regions in Wales.

SP prevalence across all age groups including both male and female participants matched against all variables appears in Table 1. The overall prevalence of SP in Wales was 4.95% (CI: 4.74%–5.17%) with a total of 847 males (4.92%, CI: 4.60%–5.24%) and 1082 females (4.98%, CI: 4.69%–5.27%) in the cohort reporting SP (Table 1). The prevalence of SP mapped by individual Welsh local authority is illustrated in Figure 2, where Flintshire exhibited the highest prevalence (6.47%, CI: 5.36%–7.57%) and Carmarthenshire with the lowest (3.85%, CI: 2.87%–4.83%).

### 3.3 Age

**FIGURE 3:**
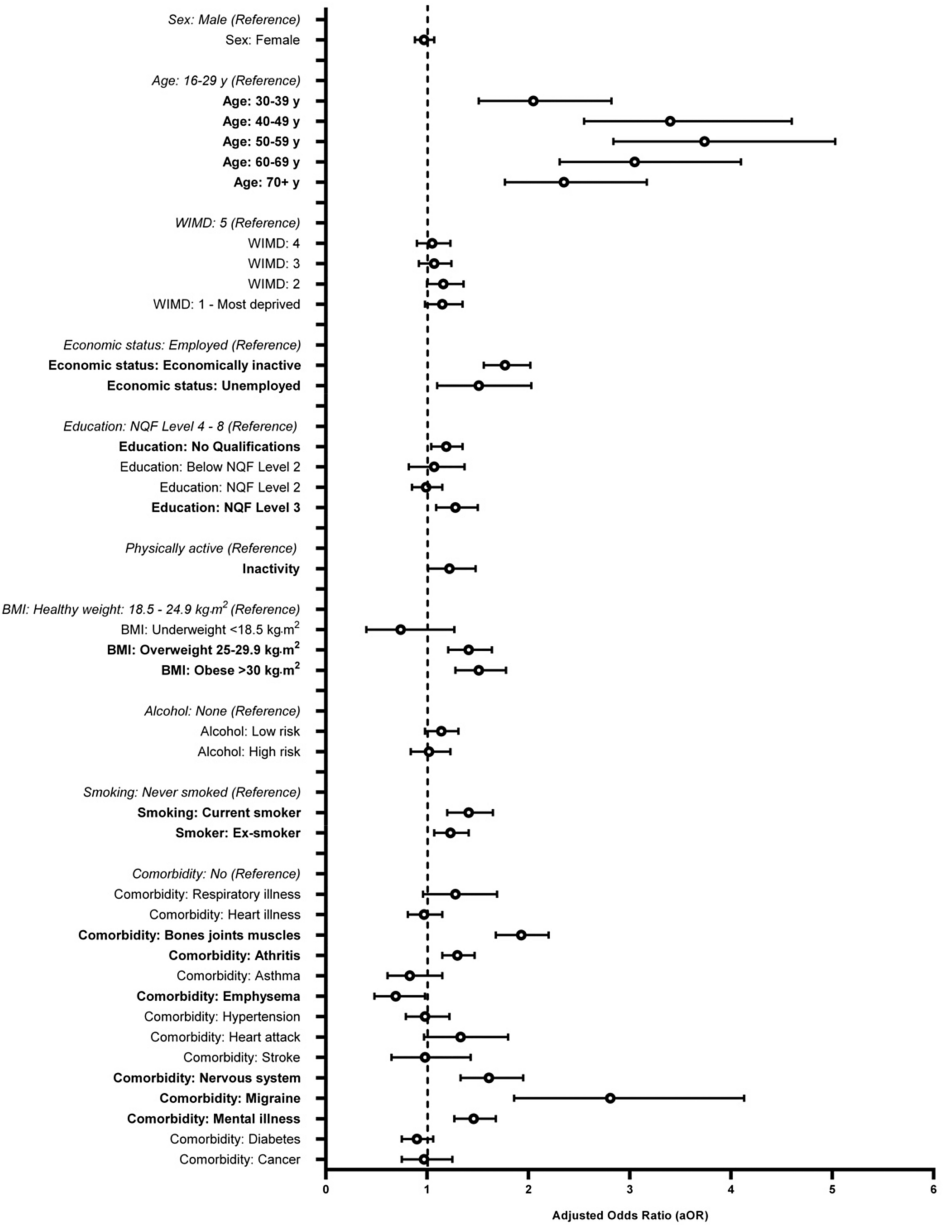
Fully adjusted odds ratios illustrating spinal pain vs. association of outcomes (determinants).

The age group with the highest prevalence of SP was in the 70+ years subgroup for both males (5.44%, CI: 4.82%–6.07%) and females (5.44%, CI: 4.82%–6.07%). The strength of association between age and SP reached its peak at 50–59 years of age and decreased slightly at 60–69 years and 70+ years. These associations were observed using a fully adjusted logistic regression model as illustrated in Figure 3. The following aOR [95%CI], *p* = <0.001 were found for 30–39 years (2.05, [1.51–2.82]); 40–49 years (3.40, [2.55–4.60]); 50–59 years (3.74, [2.84–5.03]); 60–69 years (3.05, [2.31–4.10]); 70+ years (2.35, [1.77–3.17]).

### 3.4 Biological sex

**FIGURE 4:**
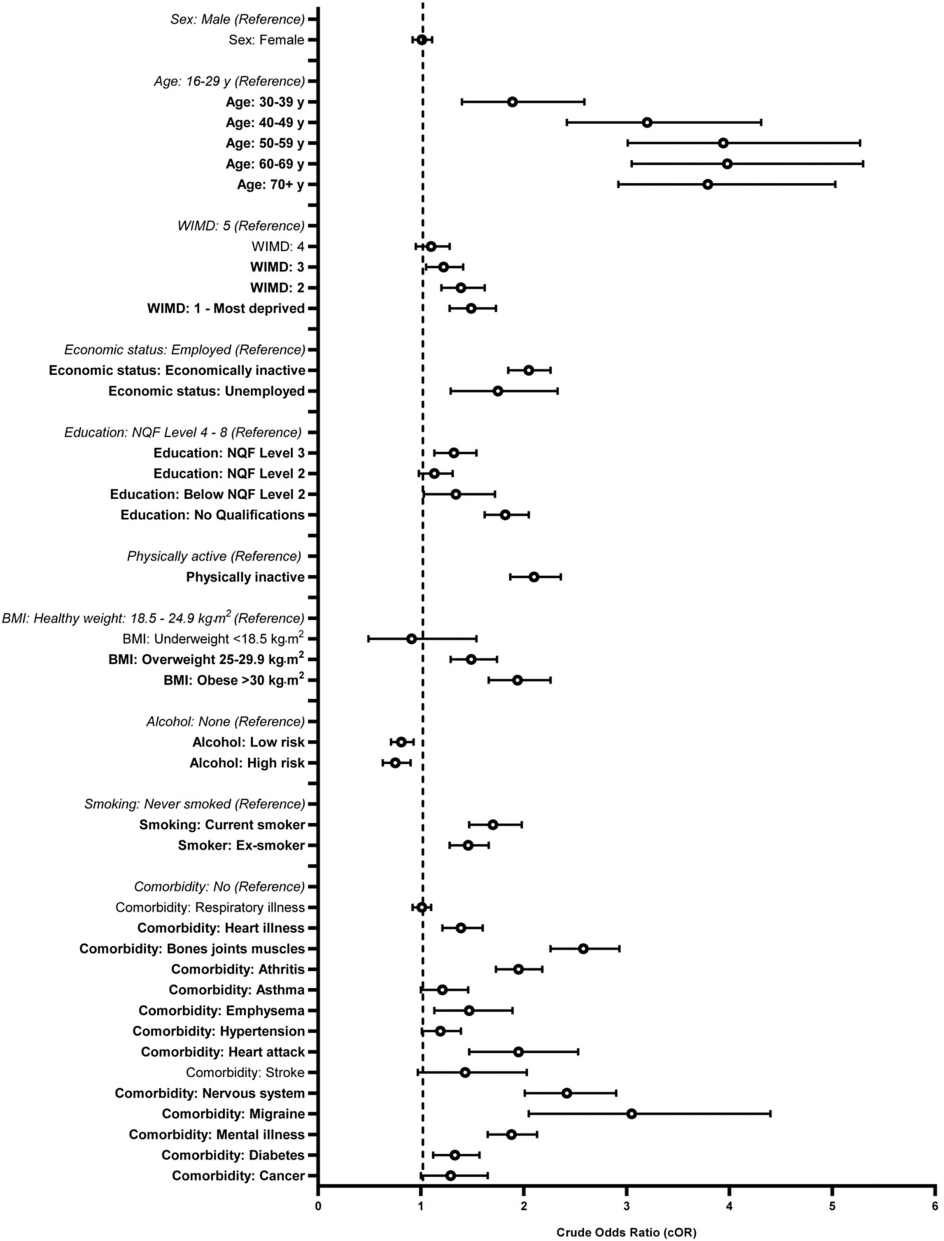
Crude (unadjusted) odds ratios illustrating spinal pain vs. association of outcomes (determinants).

There were no associations between sex and the prevalence SP as highlighted by fully adjusted or crude analyses (Figures 3 and 4 respectively).

### 3.5 Comorbidities

Population counts for comorbidities are provided in Table 1 and include the following (calculated population percentages): cancer (2.7%), diabetes (6.5%), mental illness (9.6%), migraine (0.6%), nervous system (3.2%), stroke (1.2%), heart attack (1.7%), hypertension (8.2%), emphysema (2.4%), asthma (5.5%), arthritis (12.4%), bones/joints/muscles (7.1%), heart illness (16.8%), respiratory illness (9.1%), musculoskeletal illness (21.4%), musculoskeletal limiting (19.2%), obese (13.4%), overweight (20.5%) and underweight (1.0%). Associations wth SP were confirmed for: mental illness (aOR = 1.42, CI = 1.23, 1.64, *p* = <0.001), migraine (aOR = 2.73, CI = 1.80, 4.01, *p* = <0.001), nervous system (aOR = 1.61, CI = 1.33, 1.95, *p* = <0.001), emphysema (aOR = 0.69, CI = 0.48, 0.98, *p* = 0.039), arthritis (aOR = 1.30, CI = 1.15, 1.47, *p =* <0.001), bones/joints/muscles (aOR = 1.93, CI = 1.68, 2.20, *p =* <0.001), BMI overweight (aOR = 1.41, CI = 1.21,1.64, *p* < 0.001) and BMI obese (aOR = 1.51, CI = 1.28, 1.78, *p* < 0.001) (Figure 3).

### 3.6 Lifestyle

Population counts for lifestyle factors are provided in Table 1 and comprise of the following (calculated as population percentages): physical inactivity (30.1%), ex‐smoker (19.9%), current smoker (11.2%), high‐risk alcohol (11.4%), low‐risk alcohol (35.8%). Physical inactivity responses revealed that 30% of respondents (*n* = 11,744) were recorded as inactive and of this group 6.8% reported SP (*n* = 802) (Table 1). Associations with those reporting SP were confirmed for: including: physical inactivity (aOR = 1.22, CI = 1.01, 1.48, *p* = 0.040), current smoker (aOR = 1.41, CI = 1.20, 1.65, *p* = <0.001), ex‐smoker (aOR = 1.23, CI = 1.07, 1.41, *p* = 0.003).

#### 3.6.1 Socio‐economic status

Associations with those reporting SP were confirmed for: those with no educational qualifications (aOR = 1.19, CI = 1.04, 1.35, *p* = 0.009), in addition to those with the highest educational qualifications (aOR = 1.34, CI = 1.08, 1.64, *p* = 0.006), unemployed (aOR = 1.51, CI = 1.10, 2.03, *p* = 0.008) and economically inactive (aOR = 1.77, CI = 1.56, 2.02, *p =* <0.001) (Figure 3).

## 4 DISCUSSION

The current study sought to establish a better understanding of SP prevalence and corresponding links to cardiovascular determinants albeit constrained exclusively to the Welsh population. Higher SP prevalence was associated with older age, mental and physical illness, lower educational attainment, and areas of greater deprivation. Crucially and as anticipated, SP was associated with modifiable lifestyle factors such as smoking, alcohol consumption, and physical activity. While this cross‐sectional analysis does not imply causation, a better understanding of the relationship between those reporting SP and established determinants can help to inform clinical practice and advise social policy on the scale of the health inequalities that exist in Wales. Identifying these marginalized groups provides an opportunity to formulate strategies that incorporate appropriate evidence‐based interventions (Lyons et al., 2023).

### 4.1 Spinal pain prevalence

The prevalence of SP reported in this investigation (5.0%) was lower compared to the global age‐adjusted prevalence estimates for back pain (7.5%) reported in the 2021 Global Burden of Disease study (GBD 2021 Low Back Pain Collaborators, 2023). In the Welsh population, a previous study in 2013 utilizing the WHS reported a higher prevalence of SP (39.1%), taking into account those reporting both acute (31.5%) and chronic (13.4%) pain (Jonsdottir et al., 2019). This is considerably higher than we have reported and compared to global estimates, which may be explained by the nature of the survey questions used at the time and the methodological differences between surveys. In particular, the Welsh Health Survey (2012) classified acute back pain as untreated back pain in the past 12 months and chronic pain as back pain currently being treated, which may have overestimated SP prevalence (Jonsdottir et al., 2019), whereas the current NSWD analysis employed a general health question to identify specific conditions that were affecting participants' overall health.

### 4.2 The aging spine

As anticipated, our findings demonstrate that SP prevalence increased with age, corroborating previous research within Wales (Jonsdottir et al., 2019) and globally (Briggs et al., 2016; Wu et al., 2020). The aging spine has long been associated with osteoarthritis and musculoskeletal degeneration, increasing susceptibility to damage from longstanding mechanical loads (Lindsey & Dydyk, 2023). Due to functional declines in the integrity of the spine with age, the transmission of load‐bearing forces through the spinal structures (i.e., intervertebral discs, facet joints) is altered and chronic pain can develop due to sensitisation of nociceptors over time resulting in central sensitisation (Laplante & DePalma, 2012). The peak incidence of low back pain appears to be between 80 and 89 years decreasing marginally into the next decade (Wu et al., 2020). Although we did not stratify by this specific age group, our findings support this with those aged 70+ years presenting with the highest prevalence of spinal pain, indicating that SP seems to get worse up to a specific age and then decreases depending on the study. While still associated with SP prevalence, in those aged 60–69 and 70+ years, there was a lower adjusted odds ratio in relation to SP compared with those aged 50–59 years. This contradicts the notion of a linear relationship between SP and age; however, these findings are not novel. A systematic review indicated that low back pain prevalence peaks during middle‐age (40–69 years) (Hoy et al., 2012) and the reduction thereafter may be attributed to the positive impact of retirement, namely, less manual labour/time spent at a desk and potentially increased physical activity (Hanna et al., 2019). Those who retired from physically demanding jobs, high stress work environments or were unhappy in their jobs, seem to experience an increase in health and well‐being in retirement (Henning et al., 2016).

### 4.3 Biological sex

Although we failed to observe sex‐specific differences in spinal pain prevalence, it should be noted that recent evidence has indicated that females exhibit a higher age‐standardized prevalence of SP compared to males globally (Safiri et al., 2021), a trend that has been reported in many different populations (Hanna et al., 2019; Henning et al., 2016; Laplante & DePalma, 2012; Lindsey & Dydyk, 2023). There may be many reasons for this outcome, which is beyond the scope of this study, but females seem to exhibit more determinants associated with pain prevalence (Booth et al., 2017; Kohl et al., 2012; Santos et al., 2023). However, a previous study of World Health Surveys found that females, when asked open questions, self‐report significantly poorer health than males and this difference is smaller in high‐income European countries (Boerma et al., 2016), which may contribute to the lower sex‐specific difference from the NSWD than observed in global surveys.

### 4.4 Physical inactivity

Physical inactivity is regarded as the fourth leading cause of death worldwide (Kohl et al., 2012) and remains a significant socioeconomic burden (Santos et al., 2023). Our study identified a markedly reduced physical activity in Wales, indicating that 30% of adults performed less than 30 min of activity in the previous week and was strongly associated with SP. This is concerning given the association between reduced physical activity levels and the development of a range of chronic health conditions including cardiovascular disease and the risk of neurodegenerative diseases including dementia (Booth et al., 2017; Ding et al., 2016; Hamer & Chida, 2009). Given the well‐established role of physical activity in promoting health and longevity particularly among the older population (Ciumărnean et al., 2022; D'Onofrio et al., 2023; Langhammer et al., 2018), this further emphasizes the pivotal role of physical activity initiatives on improving quality of life throughout the lifespan (Kang et al., 2020; Karimi et al., 2022).

### 4.5 Unhealthy behaviors; the impact of smoking and alcohol consumption

First, our findings demonstrate current and previous smoking status to be strongly associated with respondents reporting SP, in accordance with prior research (John et al., 2006; Palmer et al., 2003; Smuck et al., 2020). Beyond the acute analgesic effect of nicotine, long term exposure is associated with increased pain sensitivity via desensitization of nicotinic acetylcholine receptors (Iida et al., 2022). Secondly, we found alcohol intake to not be an associated determinant for SP following our adjusted analysis. Interestingly, a previous systematic review demonstrated a moderate reduction in reported pain and risk of chronic pain at lower alcohol doses with high doses indicating a global absence of association (Karimi et al., 2022). When considering knee osteoarthritis in the absence of pain, alcohol intake was found to be positively linked (Kang et al., 2020). Similarly, it has also been observed that moderate alcohol use is associated with positive pain‐related outcomes, whereas excessive alcohol abuse was associated with negative pain‐related outcomes (Zale et al., 2015).

### 4.6 Comorbidities

Patients with SP exhibit multimorbidities that collectively reflect poor health (Rafn et al., 2023). As anticipated, our results demonstrate a strong association between being overweight/obese and SP where individuals with a higher BMI have an increased risk of SP. Other studies have found a similar strong association and that these individuals are also more likely to seek additional health care services (da Cruz Fernandes et al., 2018; Shiri et al., 2010). We also observed SP and migraine headache to coexist in many individuals, consistent with the available literature (Al‐Khazali et al., 2022; Vivekanantham et al., 2019; Yoon et al., 2013), although caution should be shown due to the relatively small proportion of people with migraine (*n* = 31). The trigeminocervical convergence has been considered as a possible mechanism, with convergence of cervical and cranial nociceptive afferents and sensitization of trigeminocervical neurones (Piovesan et al., 2003). Other suggested biological mechanisms include calcitonin gene‐related peptide (CGRP) as a potential neuromodulator in these pain syndromes (Schou et al., 2017). Nitrosative and oxidative stress in platelets has also been shown to be elevated in migraine patients, especially during attacks (Yilmaz et al., 2007) and in SP conditions (Inanır et al., 2013). In addition there is central sensitisation whereby an increased nociceptive response exists in the central nervous system to sub‐threshold afferents (Harte et al., 2018). It should be noted that chronic pain, particularly SP, can be associated with various cardiac events including, myocardial infarction, stroke and cardiovascular death independent of known cardiovascular determinants (Fernandez et al., 2016; Rönnegård et al., 2022). Similarly, there exists an association between respiratory illness and spinal pain, where we observed emphysema as a confounding factor for SP, comparable to recent evidence that demonstrated chronic obstructive pulmonary disease (COPD) as a determinant for the development of persistent low back pain (Chen et al., 2017).

When considering psychological factors and spinal pain, the fully adjusted regression model reported mental illness to be significantly associated with spinal pain. This is not surprising as it has long been recognized that the development of persistent SP is strongly associated with depression, anxiety, catastrophizing, and low self‐efficacy (Hartvigsen et al., 2018; Yang et al., 2023), which are also factors considered intermediate in the pathway between experiencing neck or back pain and developing long term disability (Lee et al., 2015). Similar psychological symptoms are also present in other chronic pain conditions such as fibromyalgia and osteoarthritis, suggesting common underlying pathophysiologic mechanism of central sensitisation (Aoyagi et al., 2019; Harte et al., 2018). Concurrent pain in non‐spinal locations was also identified as significant determinant for SP in our study population. Multisite pain is very common among older adults and often associated with persistent back pain, pain severity, anxiety, and depression, and fall risk among older adults (de Luca et al., 2023; Hartvigsen et al., 2013).

### 4.7 Socio‐economic status, deprivation, and education

The Welsh Index of Multiple Deprivation (WIMD) is the Welsh Government's official measure of identifying relative deprivation for small areas of Wales, taking into account the income, employment, health, education, housing, and physical environment of individuals in each lower super output area (Bandyopadhyay et al., 2023). The primary purpose of the WIMD is to provide evidence to inform a variety of decisions about funding and services for local areas. While the results of the univariable regression analysis demonstrated a significant association for SP respondents residing in the most deprived WIMD areas (quintiles 1–3), there were no significant associations following the adjusted analysis. The social determinants of health are known to impact SP and there is clear evidence that tolerating pain is disproportionately harder for those living in deprived areas and with low quality of life (Rassu et al., 2021).

Lower educational attainment has previously been associated with SP, especially when coupled with low‐income (Hartvigsen et al., 2018; Ikeda et al., 2019; Karran et al., 2020). In support, we demonstrated those with no educational qualifications had an increased risk of SP. Individuals with a low educational attainment are more likely to work a manual job for an extended period due to the resultant lack of job opportunities (Lacey et al., 2013) and more unlikely to receive medical treatment (Buchbinder et al., 2020). Accordingly, low educational attainment is also an important predictor for chronicity (Shmagel et al., 2016) and likely impacts the severity of pain intensity reported due to lack of pain management knowledge (Köppen et al., 2018). Low socioeconomic status and low education levels are also linked with a higher prevalence of obesity, smoking, sedentary behavior and alcohol intake, raising public health concerns and placing healthcare providers at the interface to make every patient encounter count (Witkam et al., 2021).

### 4.8 Limitations

The NSWD and the WDSD utilized in this study are highly regarded for obtaining useful information on population health in Wales given the extremely large size of the population samples and associated data collected over a period of several years. This provides the opportunity to explore the relationship between a variety of health determinants including demographic, socioeconomic and lifestyle factors that provide an overall picture of the health status of the Welsh population. However, the covariates explored in this study were limited to what was collected in the NSWD and therefore, there are unknown factors that we could not account for, such as genetics and detailed family medical history. What could be considered both a strength and limitation for the study is the use of open ended health questions employed by the NSWD asking respondents to spontaneously focus on conditions that were affecting them at the time of the survey, and had to be expected to last for 12 months of more, rather than completing an extensive checklist of health conditions (Jonsdottir et al., 2019). This difference in approach may therefore account for the lower prevalence of SP observed in the NSWD and reported in this analysis. Furthermore, it has been suggested that the use of checklists in healthcare surveys may be inconsistent due to the lack of standardized methodologies (93). In addition, we didn't find any significant differences in prevalence between males and females, which may be due to the limitation of the respondents to a survey of this nature and/or due to an unrepresentative sample or responder/survivor bias, which contradicts the higher prevalence of SP in females reported across the globe.

### 4.9 Conclusions

The prevalence of SP in Wales established in this study was estimated at 5.0%. While this is lower than estimated global prevalence averages and historical estimates for Wales, the study has demonstrated that SP is closely associated with comorbidities, socio‐economic status, educational attainment, and modifiable lifestyle factors. These results can inform public health action to encourage prevention strategies by promoting protective lifestyle factors, and interventional strategies by reducing harmful modifiable factors identified. These data can also inform healthcare providers to consider strategies to manage patients with SP and the most important determinant, considering some of the public health issues related to this common clinical presentation, along with promoting various services through appropriate signposting for better patient outcomes.

## AUTHOR CONTRIBUTIONS

H.T.E. and I.W.F. conducted the data extraction from the NSWD database and performed the statistical analysis. All authors were involved in the drafting of the manuscript. D.C.B., B.S.S., H.T.E., I.W.F. and D.M.B. critically revised the manuscript and all revisions thereof. All authors contributed to and approved the final version of the manuscript.

## FUNDING INFORMATION

This study was funded by the Royal College of Chiropractors and the European Council on Chiropractic Research Excellence (ECCRE). Damian M. Bailey is supported by a Royal Society Wolfson Research Fellowship (WM170007).

## CONFLICT OF INTEREST STATEMENT

Damian M. Bailey is Editor‐in‐Chief of Experimental Physiology, Chair of the Life Sciences Working Group, member of the Human Spaceflight and Exploration Science Advisory Committee to the European Space Agency and member of the Space Exploration Advisory Committees to the UK and Swedish Space Agencies. Damian M. Bailey is also a member of the National Cardiovascular Network for Wales and South‐East Wales Vascular Network and affiliated to Bexorg, Inc. (USA) focused on the technological development of novel biomarkers of cerebral bioenergetic function and structural damage in humans.

## Data Availability

The raw data contained within the NSWD are available in linked anonymised format via the SAIL Databank, following permission from the Welsh Government and the SAIL Information Governance Review Panel. The data included within this study are available with additional permission from the corresponding author upon reasonable request.

## APPENDIX A

**TABLE A1:** Odds ratios and *p* values for the univariable and multivariable logistic regression of spinal pain, showing the reference categories.

	*N*	*Univariable analysis*		*Multivariable analysis*	
		Crude OR (95% CI)	*p* value	Adjusted OR (95% CI)	*p* value
Sex					
Male	38,954	Reference Category		Reference Category	
Female		1.01 (0.92–1.11)	0.789	0.97 (0.88–1.07)	0.508
Age (years)					
16–29	38,954	Reference Category		Reference Category	
30–39		1.89 (1.40–2.59)	**<0.001**	2.05 (1.51–2.82)	**<0.001**
40–49		3.2 (2.42–4.31)	**<0.001**	3.4 (2.55–4.60)	**<0.001**
50–59		3.94 (3.01–5.27)	**<0.001**	3.74 (2.84–5.03)	**<0.001**
60–69		3.98 (3.05–5.30)	**<0.001**	3.05 (2.31–4.10)	**<0.001**
70+		3.79 (2.92–5.03)	**<0.001**	2.35 (1.77–3.17)	**<0.001**
**WIMD**					
5—Least deprived	38,954	Reference Category		Reference Category	
4		1.10 (0.95–1.28)	0.203	1.05 (0.90–1.23)	0.510
3		1.22 (1.05–1.41)	**0.010**	1.07 (0.92–1.24)	0.407
2		1.39 (1.20–1.62)	**<0.001**	1.16 (1.00–1.36)	0.056
1—Most deprived		1.49 (1.28–1.73)	**<0.001**	1.15 (0.98–1.35)	0.084
Education					
NQF levels 4–8	38,954	Reference Category		Reference Category	
NQF level 3		1.32 (1.13–1.54)	**0.001**	1.28 (1.09–1.50)	**0.002**
NQF level 2		1.13 (0.98–1.31)	0.093	0.99 (0.85–1.15)	0.873
Below NQF level 2		1.34 (1.03–1.72)	**0.023**	1.07 (0.82–1.37)	0.633
No qualifications		1.82 (1.62–2.05)	**<0.001**	1.19 (1.04–1.35)	**0.009**
Economic status					
Employed	38,954	Reference Category		Reference Category	
Unemployed		1.75 (1.29–2.33)	**<0.001**	1.51 (1.10–2.03)	**0.008**
Economically inactive		2.05 (1.85–2.26)	**<0.001**	1.77 (1.56–2.02)	**<0.001**
BMI (kg/m^2^)					
Underweight (< 18.5)	38,954	0.91 (0.49–1.54)	0.756	0.74 (0.40–1.27)	0.307
Healthy weight (18.5–24.9)		Reference Category		Reference Category	
Overweight (25–29.9)		1.49 (1.29–1.74)	**<0.001**	1.41 (1.21–1.64)	**<0.001**
Obese (>30)		1.94 (1.66–2.26)	**<0.001**	1.51 (1.28–1.78)	**<0.001**
Physical activity					
Active	38,954	Reference Category		Reference Category	
Inactive		2.10 (1.87–2.36)	**<0.001**	1.22 (1.01–1.48)	**0.040**
Smoker					
Never smoked	38,954	Reference Category		Reference Category	
Ex‐smoker		1.46 (1.28–1.66)	**<0.001**	1.23 (1.07–1.41)	**0.003**
Current smoker		1.70 (1.47–1.98)	**<0.001**	1.41 (1.20–1.65)	**<0.001**
Alcohol					
None	38,954	Reference Category		Reference Category	
Low risk		0.81 (0.71–0.93)	**0.003**	1.14 (0.98–1.31)	0.084
High risk		0.75 (0.63–0.90)	**0.002**	1.02 (0.84–1.23)	0.874
Multicomorbidities					
Cancer*	38,954	1.29 (1.00–1.65)	**0.045**	0.97 (0.75–1.25)	0.838
Diabetes*		1.33 (1.12–1.57)	**0.001**	0.9 (0.75–1.06)	0.214
Mental illness*		1.88 (1.65–2.13)	**<0.001**	1.46 (1.27–1.68)	**<0.001**
Migraine*		3.05 (2.05–4.40)	**<0.001**	2.81 (1.86–4.13)	**<0.001**
Nervous system*		2.42 (2.01–2.90)	**<0.001**	1.61 (1.33–1.95)	**<0.001**
Stroke*		1.43 (0.97–2.03)	0.056	0.98 (0.65–1.43)	0.925
Heart attack*		1.95 (1.47–2.53)	**<0.001**	1.33 (0.97–1.80)	0.068
Hypertension*		1.19 (1.01–1.39)	**0.029**	0.98 (0.79–1.22)	0.854
Emphysema*		1.47 (1.13–1.89)	**0.003**	0.69 (0.48–0.98)	**0.039**
Asthma*		1.21 (1.00–1.46)	**0.041**	0.83 (0.61–1.15)	0.263
Arthritis*		1.95 (1.73–2.18)	**<0.001**	1.3 (1.15–1.47)	**<0.001**
Bones, joints, muscles*		2.58 (2.26–2.93)	**<0.001**	1.93 (1.68–2.20)	**<0.001**
Heart illness*		1.39 (1.24–1.55)	**<0.001**	0.97 (0.81–1.15)	0.704
Respiratory illness*		1.39 (1.21–1.60)	**<0.001**	1.28 (0.96–1.69)	0.082

Abbreviations: BMI, Body Mass Index; NQF, National Qualifications Framework; WIMD, Welsh Index of Multiple Deprivation.

*Note*: *p* values <0.05 have been presented in bold.

* Reference category is “No.”
